# Self-care for head and neck cancer survivors with lymphedema and fibrosis: study protocol for a randomized controlled trial

**DOI:** 10.1186/s13063-019-3819-0

**Published:** 2019-12-27

**Authors:** Jie Deng, Mary S. Dietrich, Barbara Murphy

**Affiliations:** 1 School of Nursing, University of Pennsylvania, 418 Curie Boulevard, Philadelphia, PA 19104-4217 USA; 2 School of Nursing, Vanderbilt University, Nashville, TN USA; 3 Vanderbilt-Ingram Cancer Center, Vanderbilt Medical Center, Nashville, TN USA

**Keywords:** Head and neck cancer, Lymphedema, Fibrosis, Self-care, Self-management, Survivorship

## Abstract

**Background:**

Head and neck cancer (HNC) patients are at high risk for developing lymphedema and fibrosis (LEF) following cancer treatment. Once HNC patients develop LEF, they need to conduct life-long self-care to slow LEF progression and reduce associated symptom burden and functional deficits. Data demonstrate that inadequate LEF self-care may be a potentially remediable issue. The objective of this study is to explore the feasibility and preliminary efficacy of an Information-Motivation-Behavioral (IMB) Skills model-driven self-care program (SCP) to improve LEF management and reduce LEF-related symptom burden and functional impairments.

**Methods/design:**

This is a three-arm, prospective, randomized controlled clinical trial to compare: Group 1 – Usual Care, Group 2 – Usual Care Plus LEF-SCP, and Group 3 – Usual Care Plus LEF-SCP Plus Follow-Up. Participants will be HNC survivors aged > 18 years of age, who meet predefined inclusion and exclusion criteria. A sample size of 75 participants is targeted. Interventions will be provided by trained staff. The study assessments for all groups will take place at five points: study entry then 3, 6, 9, and 12 months post enrollment. Outcome measures include: (1) feasibility (barriers to implementation, safety, and satisfaction) of the proposed intervention; (2) self-efficacy and adherence to self-care; and (3) preliminary efficacy (LEF progression, symptom burden, and functional status) of the proposed intervention.

**Discussion:**

This will be the first study to evaluate the feasibility of a LEF-SCP in the HNC population and its impact on self-efficacy and adherence. Furthermore, it will evaluate the potential benefit of routine follow-up on adherence and fidelity to the self-care protocol. We expect that the trial will provide evidence supporting the feasibility of a LEF self-care program. In addition, we anticipate that preliminary data will support improved outcomes including increased adherence and fidelity, and decreased LEF-associated symptoms.

**Trial registration:**

ClinicalTrials.gov, a service of the US National Institute of Health (NCT 03030859). Registered on 22 January 2017.

## Background

The incidence of head and neck cancer (HNC) has increased rapidly due to the epidemic of human papillomavirus (HPV)-associated oropharyngeal carcinoma [1, 2]. Patients with HPV-associated HNC have a wider age range, thus a cohort of patients are young or middle-aged. Because smoking is not a prerequisite, patients with HPV-associated HNC tend to be healthier with a longer life expectancy. Finally, HPV-associated HNC is highly responsive to therapy and has improved outcomes compared to smoking-related HNC [3, 4]. Thus, the number of HNC survivors is increasing rapidly. We now have more than half a million HNC survivors within the United States [5]. Unfortunately, treatment for many patients with locally advanced HNC requires multi-modality treatment [6] associated with numerous acute and long-term toxicities. Soft-tissue damage with resulting secondary lymphedema and fibrosis (LEF) is seen in a high percentage of survivors [7, 8].

LEF is a trans-tissue process that affects three quarters of HNC patient more than 3 months post treatment [7]. It manifests as soft tissue swelling and fibrosis [7, 8] involving external structures (e.g., face and neck) and internal structures (e.g., tongue and epiglottis) [7, 9]. The impact of LEF is profound with a resultant high symptom burden, substantial functional deficits, and diminished quality of life (QOL) [10]. Involvement of the skin leads to alerted sensation in the skin with a sense of discomfort, pressure and tightness [9, 10]. When it affects the underlying connective tissue and muscles, LEF may result in decreased range of motion, adversely impacting function [10–12]. Internal LEF may result in dysphagia, altered speech, and airway compromise [10, 11]. Finally, LEF may be associated with increased anxiety and altered body image [9, 11].

Currently, head and neck lymphedema is an incurable but manageable, chronic condition [11, 13, 14]. Data on management of HNC-associated lymphedema is limited [9, 11, 15–17]. Usual care for management of head and neck lymphedema is based on clinical experience or data from other anatomical sites of lymphedema [11]. Current usual care (standard of care) for lymphedema is complete decongestive therapy (CDT) including intensive lymphedema therapy (Phase I of CDT) with a certified lymphedema therapist, followed by long-term self-care (Phase II of CDT) administered by the patient and/or a caregiver [13, 14, 18, 19]. Optimally, lymphedema therapists would conduct routine follow-up assessments for patients with more severe soft tissue toxicities during the self-care phase. Follow-up would include assessment of fidelity to self-care practices and screening for changes in the status of the soft tissue, which would require alterations in the self-care plan. Currently, routine follow-up by therapists is not part of usual care in the United States and is not covered by most insurance companies. This becomes problematic because: (1) soft tissue changes can worsen over time, which may require a change in the care regimen; and (2) fidelity and adherence to self-care practices may diminish over time without continued follow-up. Studies exploring the role of follow-up care are needed in order to understand the impact of this therapeutic gap.

While it is generally agreed that self-care is critical for long-term management in patients with LEF, the self-care component of HNC-associated lymphedema management has not been standardized. There are no evidence-based LEF self-care programs conducted in HNC populations in the literature; thus, in clinical settings, self-care regimens may vary widely based on the experience and training of the lymphedema therapist [20]. Indeed, some HNC patients fail to receive adequate training in self-care. Studies focused on enhancing LEF self-care (e.g., establishing a standardized self-care program) in HNC patients are warranted [21].

The Information-Motivation-Behavioral (IMB) Skills model of health behavior change has been used in addressing self-care issues faced by individuals with chronic conditions. A recent systematic review assessed the applicability and effectiveness of interventions based on the IMB model for promoting behavioral changes among individuals with chronic diseases [22]. Special attention was paid to interventions focusing on self-care behaviors (e.g., heart disease self-care) and risk prevention (e.g., risky sexual behaviors) [22]. Currently, no studies have been available to evaluate self-care behavioral changes in HNC patients with LEF based on the IMB model. Recent literature reported a number of issues related to long-term lymphedema self-care in the HNC population [21]. More than half of the participants failed to adhere to self-care regimens due to lack of motivation, low self-efficacy, and lack of continued guidance [21]. Indeed, if the IMB model is successfully applied in HNC survivors with LEF, inadequate self-care may be a potentially remediable issue. Thus, the authors developed an IMB model-driven intervention to facilitate HNC survivors’ LEF self-care. Based on the IMB model, adherence to self-care regimens may be determined by the extent to which a patient: (1) understands self-care and its importance; (2) is motivated to adhere to a self-care program; and (3) has behavior skills required to ensure adherence over time [23]. The objective of this study is to conduct a three-arm pilot trial with the goal of identifying the optimal regimen for LEF self-care for a future definitive Phase III trial. If it is successful, the IMB model-driven self-care intervention could be utilized by lymphedema therapists as the basis for training patients and/or caregivers, thereby improving long-term outcomes in the HNC population. Aim 1 of this study is to determine the feasibility of implementing a LEF self-care program (SCP) with or without routine follow-up in a sample of HNC survivors with LEF. Aim 2 is to obtain preliminary data regarding the impact of the SCP with or without follow-up on self-efficacy and adherence to self-care. Aim 3 is to obtain preliminary data regarding the impact of the SCP with or without follow-up on outcomes of interest including LEF progression, symptom burden and functional status.

## Methods/design

**Fig. 1:**
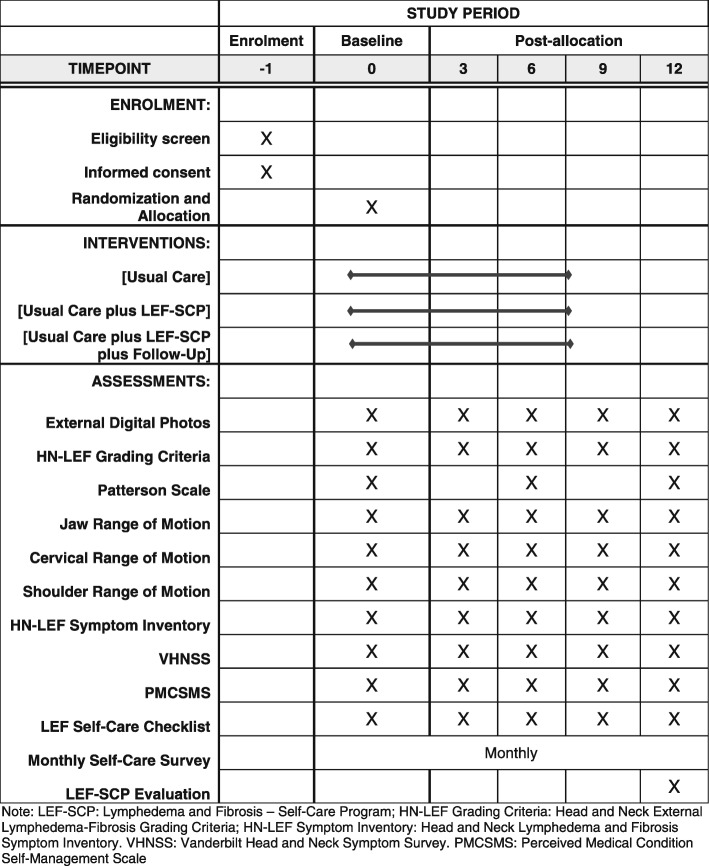
Standard Protocol Items: Recommendation for Interventional Trials (SPIRIT) Figure

The study was approved by the Institutional Review Board at the University of Pennsylvania. Recruitment will be conducted at the Head and Neck Cancer Clinics in the University of Pennsylvania Health System. The protocol has been developed in accordance with the Standard Protocol Items: Recommendations for Interventional Trials (SPIRIT) guidelines for interventional trials [24]. The SPIRIT Checklist and Figure are given in Additional file 1 and Fig. 1, respectively.

### Overview of study design

This study is a three-arm, prospective, randomized controlled clinical trial to compare: (1) Usual Care, (2) Usual Care Plus LEF-SCP, and (3) Usual Care Plus LEF-SCP Plus Follow-Up. Outcome measures include: (1) feasibility (barriers to implementation, safety, and satisfaction) of the LEF-SCP (Aim 1); (2) self-efficacy and adherence to self-care (Aim 2); and (3) LEF progression, symptom burden, and functional status (Aim 3). The study assessments will take place at five points in time, enrollment, 3, 6, 9, and 12 months post enrollment.

### Inclusion criteria

Individuals will be recruited into the study if they meet the following criteria: (1) post - HNC primary treatment; (2) no evidence of cancer; (3) no more than 6 months after completion of initial lymphedema therapy for head and neck lymphedema; (4) > 18 years of age; (5) the ability to understand English in order to complete questionnaires; (6) ability to complete the onsite training and home self-care activities for LEF management; and (7) ability to provide informed consent.

### Exclusion criteria

Individuals will be excluded if they have any of the following medical conditions that would prohibit the safe implementation of self-care of LEF: recurrent or metastatic cancer; any other active cancer; acute infection; congestive heart failure; renal failure; cardiac or pulmonary edema; sensitive carotid sinus; severe carotid blockage; or uncontrolled hypertension.

### Recruitment

A minimum of five participants will be recruited per month. Our previous studies in a similar patient population have achieved recruitment goals in a timely manner. This was largely due to the research team’s dedication, support from attending physicians, and a concerted effort on the part of investigators to minimize patient burden. The volume of patients at the Head and Neck Cancer Clinics in the University of Pennsylvania Health System is sufficient to complete this trial without additional sites. The recruitment procedures used will be identical to those used successfully in our prior studies, such as screening at Head and Neck Cancer Clinics. All direct patient recruitment activities will be conducted at the Head and Neck Cancer Clinics where private rooms are available to be used for conducting clinical research projects.

### Randomization and allocation

Participants will have provided written informed consent, been screened, and completed all study baseline measures prior to study group randomization. Seventy-five participants will be randomized via the use of a computer-generated, permuted block program, developed and executed by the study statistician, to one of the three study groups (Usual Care, Usual Care Plus LEF-SCP, Usual Care Plus LEF-SCP Plus Follow-Up) at a 1:1:1 allocation ratio (*n* = 25 per group).

### Blinding

Due to the nature of the interventions, neither participants nor staff can be blinded to the allocation status of the participants.

### Content and delivery of interventions

#### Usual Care group

After completion of the baseline measures, Usual Care participants will receive usual care only, without any additional interventions.

#### Usual Care Plus LEF-SCP group

After completion of the baseline measures, Usual Care Plus LEF-SCP participants will be scheduled for the LEF-SCP training. The training includes three, weekly, face-to-face sessions composed of a 1-h motivational interviewing (MI) session plus a 1-h LEF self-care training session. After completion of the LEF-SCP training, participants will be given the LEF self-care educational manual. Participants with Internet access will be provided a safe website link to watch the self-care videos. Participants without Internet access will be given the Digital Versatile Discs (DVDs) and a DVD player for watching the videos. Participants will be asked to review the manual and videos monthly or more frequently as needed.

#### Usual Care Plus LEF-SCP Plus Follow-Up group

After completion of the baseline measures, participants in the Usual Care Plus LEF-SCP Plus Follow-Up group will follow the same procedures as the Usual Care Plus LEF-SCP group. *In addition*, however, participants in this group will be scheduled to meet with the study lymphedema therapist for 3-, 6-, and 9-month LEF follow-ups. Please note that these follow-ups will take place after completion of 3-, 6-, and 9-month follow-up data collection. Each LEF follow-up visit with the lymphedema therapist will take approximately 1 h. The lymphedema therapist will check participants’ LEF status, adjust the LEF self-care plan if needed, make new recommendations about LEF self-care, and review and address any potential issues and questions that participants may have about LEF self-care. The lymphedema therapist will document the suggestions given to each participant.

### Measures

#### Patient characteristics

*The Demographic Form*: this form will be used to collect the following participant data: date of birth, gender, race, ethnicity, education, marital and employment status, area of residence, medical history, alcohol use, tobacco use, and dietary habits. *The HNC Clinical Form*: a medical chart review will be conducted to collect data on the diagnosis date of HNC, primary site, stage, type and dates of treatment, and complications. *The LEF Treatment Form*: the form will be used to collect date of diagnosis of lymphedema and/or fibrosis and treatment history.

#### Feasibility measures

*The Recruitment Log* includes the number screened, number recruited, and barriers to recruitment. *The Implementation Log-MI Session* will be used by the study MI instructor to document barriers of implementing MI sessions. *The Implementation Log-LEF Self-Care Training Session* will be used by the study lymphedema therapist to document the barriers of implementing LEF self-care training sessions. *The Common Terminology Criteria for Adverse Events (CTCAE version 5.0)* will be used to document any adverse events [25]. *The LEF-SCP Evaluation (Satisfaction)* will be completed by participants in the two LEF-SCP study groups after their 12-month post-enrollment assessment has been completed.

#### Self-efficacy measure

*The Perceived Medical Condition Self-Management Scale (PMCSMS)*: an 8-item generic scale that can be adapted to any specific medical condition (e.g., LEF), will be used to assess self-efficacy in self-managing LEF (Cronbach’s alpha 0.84) [26–28].

#### Self-care adherence measures

*The LEF Self-Care Checklist*: the form asks participants to complete a checklist of LEF self-care activities they have performed during the past 7 days. Participants will complete this checklist at the baseline, 3-, 6-, 9-, and 12-month follow-ups. *Monthly Self-Care Survey*: participants will receive an email survey during the last week of each month during the study follow-up. They will be asked to answer four to five brief questions about their adherence to the LEF self-care activities during the past 7 days (e.g., Have you conducted daily self-care for your lymphedema and/or fibrosis during the past 7 days? Response: Yes or No).

#### LEF evaluation

*Digital photographs* of the head and neck region will be obtained at baseline and all follow-up visits. A standardized technique for photography has been established by the team in order to ensure constancy. *Head and Neck External Lymphedema-Fibrosis (HN-LEF) Grading Criteria* will be used to document participants’ external LEF status. Our previous study supports the scale with content/face validity and good interrater reliability (e.g., kappa = 0.752, *p* < 0.001) [8]. *The Patterson Scale* is a validated measure for documenting internal edema in the pharynx and larynx. The scale has good intrarater reliability (weighted kappa = 0.84) and moderate interrater reliability (weighted kappa = 0.54) [29]. This scale will be completed by participants’ primary physicians as part of standard of care for HNC patients.

#### Symptom burden

*Head and Neck Lymphedema and Fibrosis Symptom Inventory*: a patient-reported outcome measure developed specifically to assess lymphedema symptoms in HNC patients. Content and face validity of the tool was reported during the development and preliminary testing of the tool. The tool has 58 items (Cronbach’s alpha for five subscales: 0.80–0.97) [30, 31]. *Vanderbilt Head and Neck Symptom Survey Version 2.0 (VHNSS v2.0)*: a validated measure of HNC treatment-related symptoms, 50 items (Cronbach’s alpha = 0.94) [32–34].

#### Functional status

*Jaw Range of Motion Scale*: a validated and reliable device for measuring jaw range of motion [35, 36]. *Cervical Range of Motion Device*: a validated and reliable device for measuring the degree of neck movement [37]. *Goniometry*: a validated and reliable instrument for measuring active and passive shoulder range of motion [38, 39].

### Statistical analyses

Assumptions underlying each of the proposed statistical summaries and comparative analyses will be evaluated. Transformation of continuous data distributions will be completed as necessary to meet those assumptions. Randomly missed responses to items within assessment tools will be handled via protocols specified by the instrument developers. We expect that entire assessments (e.g., 9 months post) will not be missing at random, thus imputation of missing data is not planned. While this study is a preliminary study of efficacy and effect sizes, a maximum alpha level of 0.05 will be used for the statistical tests.

#### Feasibility of the LEF-SCP

Descriptive statistical and graphical methods will summarize the rates of participation (recruited vs. consented), log data (e.g., sessions completed), completion of assessments throughout the study, adverse events (safety) and satisfaction data (i.e., LEF-SCP program evaluation) within the two groups assigned to LEF-SCP. Qualitative data analysis will be used to summarize the barriers to performing self-care. It is expected that ≥ 80% of participants will complete the study activities, and ≥ 80% will report acceptable levels of satisfaction with the LEF self-care, thereby demonstrating feasibility of the LEF self-care.

#### Self-efficacy and self-care adherence

Descriptive statistical and graphical methods will be used to summarize and visually inspect the PMCSMS scores (self-efficacy), the number and types of self-care activities reported on the LEF Self-Care Checklist within the three study groups throughout the study period. Mixed-effects generalized linear models that correct the standard errors for repeated assessments and incorporate appropriate distributional link functions (e.g., log-link for skewed distributions) will be used to generate estimates of the effect of the LEF-SCP on self-efficacy and rates of self-care. Within the analyses, bias-corrected estimates of the interaction of group assignment with the time of assessment (baseline, 3-, 6-, 9-, and 12-month follow-ups) on the outcome measures will provide the key effects interest (i.e., differences among the groups in the longitudinal patterns of self-efficacy and rates of adherence to self-care).

#### Preliminary efficacy of the LEF-SCP

As with the previous aim, descriptive statistical and graphical methods will be used to summarize and visually inspect the measures of LEF, symptom burden, and functional status within the three study groups throughout the study period. Mixed-effects generalized linear models with the appropriate link function for the nature of the specific outcome variable being analyzed will be used to generate estimates of the effect of the LEF-SCP on the progression of head and neck LEF, symptom burden, and function measures. Bias-corrected estimates of the interaction of group assignment with time of assessment will provide the key effects interest (i.e., differences between the groups in the patterns or slopes of changes in the outcome measures over time).

### Sample size justification

The primary purpose of this study is to inform the feasibility and potential efficacy of a tailored LEF self-care program among the population of HNC survivors with LEF. The proposed sample sizes are believed sufficient for gleaning this information. Rather than formal statistical tests of hypotheses, effect sizes generated are the most critical statistical outcome. The expected analysis sample of three groups (25 per group, total *N* = 75) will meet the critical assumptions for estimating central tendency and variability in the key outcome values, as well as for generating estimates of the effects of the LEF-SCP on self-efficacy, self-care adherence, progression of LEF, symptom burden, and functional status. Given the emphasis on effect sizes, there is no plan to adjust for the multiple measures included in this study and any conclusions regarding statistical significance will be based on a two-sided-type alpha of 0.05.

### Risks and safety monitoring

Although this is an interventional study, foreseeable risks from study participation are believed to be minimal. The key components of the LEF-SCP were developed based on the current standard of care of LEF as well as the IMB model of health behavioral change. All these key components have been used in individuals with other chronic conditions. No physical risks (e.g., worsening LEF) from implementing these components have been reported in the known current literature review. However, if study staff identify LEF-associated issues (e.g., infection, sudden increase in swelling) requiring intervention, the principal investigator (PI), study physician, and study lymphedema therapist will be notified immediately. Patients will be referred to their treating physicians and lymphedema therapist for adequately managing their LEF in a timely manner.

### Reporting of unanticipated problems

The PI, study physician, and study lymphedema therapist will review any adverse events and other unanticipated problems. The PI will report any adverse events and other unanticipated problems to the University of Pennsylvania IRB and the American Cancer Society.

### Withdrawal and discontinuation

Participants will be withdrawn from the study if they develop recurrent or metastatic cancer and any other active cancer.

### Confidentiality

All participants will be assigned a study ID number by a trained study staff member. This information will be maintained in a patient-tracking database that is not stored on the hard drive, accessible only to the study team members through a password-protected computer and link to the server at the study site, which is accessible only the study team.

## Discussion

More than half a million HNC survivors are alive in the United States [5]. Three - quarters of them are expected to experience LEF (swelling and hard tissues in the head and neck region) after their cancer and/or cancer therapy [7]. Once skin/soft tissues are involved by LEF, it requires HNC survivors to conduct life-long self-care of this chronic condition to slow its progression and reduce associated symptom burden (e.g., neck tightness) and functional issues (e.g., difficulty swallowing) [10, 13, 14, 19]. Despite the need for daily self-care, about half of the patient population does not regularly perform LEF self-care. The critical barriers hampering LEF self-care in HNC survivors include lack of needed information/knowledge, lack of motivation, and inadequate self-care skills [21]. This study will evaluate whether an IMB model-driven intervention that effectively addresses barriers of LEF self-care enhances adherence to self-care behaviors and decreases symptom burden and functional deficits in this population. It is expected that participants receiving the LEF-SCP will report improved outcomes compared to Usual Care; and that participants receiving the LEF-SCP with follow-up will report improved outcomes compared to LEF-SCP without follow-up. The proposed intervention may have the potential to improve self-care, symptom burden, functional deficits, and quality of HNC survivorship. The data will be published after the study is completed.

### Trial status

The date recruitment began: 1 June 2018.

The approximate date when recruitment will be completed: 31 May 2020.

Data are currently being collected. At the time of submission of this paper (28 April 2019), the protocol is version 2.0 (14 January 2019).

## Supplementary information

**Additional file 1:.** Standard Protocol Items: Recommendations for Interventional Trials (SPIRIT) Checklist.

## Data Availability

Not applicable.

## Abbreviations

CDT: Complete decongestive therapy
CTCAE: Common Terminology Criteria for Adverse Events
HNC: Head and neck cancer
HN-LEF Grading Criteria: Head and Neck External Lymphedema-Fibrosis Grading Criteria
HN-LEF Symptom Inventory: Head and Neck Lymphedema and Fibrosis Symptom Inventory
HPV: Human papillomavirus
IMB Skills model: Information-Motivation-Behavioral Skills model
LEF: Lymphedema and fibrosis
MI: Motivational interviewing
PMCSMS: Perceived Medical Condition Self-Management Scale
QOL: Quality of life
SCP: Self-care program
VHNSS: Vanderbilt Head and Neck Symptom Survey
